# Supplementary material for: Impingement of deposited water nanodroplets with coming nanoparticles: A molecular dynamics study
Source: PLoS One. 2026 Jan 5;21(1):e0340196. doi: 10.1371/journal.pone.0340196 (PMC12768267; doi:10.1371/journal.pone.0340196)
Supplement: Attachment — Submitted filename: Response to Reviewer 2.docx [file pone.0340196.s003.docx]

**Response to Reviewer**

Overall, the study is well-structured, and the MD simulation approach is appropriately employed to explore the effects of key parameters (e.g., particle velocity, size, wettability, and surface wettability). The phase diagrams summarizing five possible outcomes (deposition, wrapped bounce, adhesion bounce, separated bounce, and separation without bounce) effectively synthesize the experimental results and provide clear insights into the system’s behavior.

However, several minor revisions are recommended to enhance the manuscript’s quality and rigor:

Ans: First of all, thank you for your comments, which are very helpful in improving our paper. Subsequently, we tried our best to revise our manuscript and addressed your comments point-by-point. Please see the following responses. Please note that the corrections are written in red and the responses are written in blue in both this response letter and the marked-up manuscript.

1. Typos and Terminology Consistency: Correct spelling errors (e.g., "Nosadays" → "Nowadays", "swichable" → "switchable", "adhension" → "adhesion") and ensure consistent use of technical terms throughout the text.

Ans: Thank you for your valuable comment. We have rechecked the manuscript and made the necessary corrections in the revised manuscript.

2. Mechanism Elaboration: Supplement quantitative analysis of energy conversion (e.g., the ratio of kinetic energy to surface energy transformation in different dynamic processes) to deepen the understanding of underlying mechanisms.

Ans: Thank you for your valuable comment. Please see the following statement in the revised manuscript: “The progress of the collision is accompanied by energy conversion between the incoming particle and the water droplet. As shown in **Figure 2b**, the collisional process quickly reduces the particle’s kinetic energy, whose value drops to null within a very short time. On the other hand, the surface energy of the droplet increases so that it spreads upon the solid surface. The fast consumption of the particle’s kinetic energy traps it at the upper part of the deposited droplet, see its internal structure in **Figure 2a**. After *E*_s, dim_ reaches its maximum value, the droplet starts to retract and vibrate with its value rising and falling. However, the energy of the droplet can not overcome the work of adhesion, and thus, the droplet eventually forms a stable wetted state after several vibrations”

(please see page 7-8, Lines 20-22; Lines 1-6)

3. Parameter Rationale: Clarify the justification for selecting critical parameters (e.g., the deposited water droplet diameter of 8 nm and the range of nanoparticle sizes) to strengthen the study’s methodological rigor.

Ans: Thank you for your valuable comment. In MD simulations, the increase in droplets’ size can significantly consume computing resource, whereas the thermal disturbance can affect dynamic evolution if the size is too small. Therefore, the diameter of water droplets is set as 8 nm, which is appropriate for exploring impact dynamics.

4. Figure Annotation: Verify and correct formatting errors in figure captions (e.g., Figure 7(a) and 7(b) both label Δ=0.75, which may be a typo) to improve readability.

Ans: Thank you for your valuable comment. We have corrected formatting errors in the revised manuscript.

5. Comparison with Existing Work: Enhance discussions on the novelty of this study by comparing it with relevant literature, highlighting how the findings advance current knowledge in droplet manipulation.

Ans: Thank you for your valuable comment. Please see the following statement in the revised manuscript: “ In addition, we map two phase diagrams with respect to the dimensionless input kinetic energy (*E*_k, dim_), the diameter of the nanoparticle to that of the water droplet (*Δ*), and the intrinsic wettability of the surface (*θ*_sur_) to overall investigate these effects and observe all the possible outcomes. This work paves the way to understanding the progress of impingement of nanoparticles on deposited water droplets upon surfaces, which may be a good candidate for rapidly removing deposited water droplets and recovering the hydrophobicity of solid surfaces.”

(please see page 1, Lines 17-22)
